# Development and evaluation of a real-time quantitative PCR for the detection of equine infectious anemia virus

**DOI:** 10.1128/spectrum.02599-23

**Published:** 2023-10-09

**Authors:** Shuaijie Li, Kui Guo, Xuefeng Wang, Yuezhi Lin, Jinhui Wang, Yaoxin Wang, Cheng Du, Zhe Hu, Xiaojun Wang

**Affiliations:** 1 State Key Laboratory for Animal Disease Control and Prevention, Harbin Veterinary Research Institute, the Chinese Academy of Agriculture Sciences, Harbin, China; 2 WOAH Reference Laboratory for Equine Infectious Anemia, Harbin Veterinary Research Institute of Chinese Academy of Agricultural Sciences, Harbin, China; Changchun Veterinary Research Institute, Changchun, China

**Keywords:** equine infectious anemia, gag gene, tat gene, real-time quantitative PCR

## Abstract

Equine infectious anemia (EIA) has a worldwide distribution and causes severe economic losses to the equine industry. The EIA virus (EIAV) genome sequences from different countries are highly diverse, which poses a great challenge for pathogen identification with PCR. Phylogenetic analysis showed that although gag is the most conserved structural gene, it still has great genome variability. Currently, most existing PCR methods are designed based on the gag gene sequence and therefore do not cover all the viral strains, especially Asian EIAV strains. In this study, we developed a tat-gag-based real-time quantitative PCR (TG-qPCR) for the detection of EIAV by targeting the fragment between the tat and gag genes, which was relatively conserved in all the known EIAV strains. The performance of the TG-qPCR was evaluated against that of the standard qPCR (recommended by WOAH) by testing viral RNA extracted from viral supernatants of EIAV_DLV2-6_ and EIAV_UK3_, proviral DNA from peripheral blood mononuclear cells of artificially immunized horses, and virus nucleic acid from EIAV positive serum samples. The TG-qPCR assay had high specificity, sensitivity, and reproducibility. The detection limit of the TG-qPCR assay was 1 copy/reaction for both viral RNA and proviral DNA based on the Poisson distribution. Compared to the qPCR, the TG-qPCR has better inclusivity and can detect not only Asian EIAV strains but also almost all the representative EIAV strains from other continents. The above results show that the TG-qPCR assay could serve as an effective tool for the early diagnosis of clinical EIA disease.

**IMPORTANCE:**

Equine infectious anemia (EIA) has a worldwide distribution and causes significant losses to the equine industry worldwide. A reliable detection method is necessary to control the transmission of EIA virus (EIAV). Currently, most of the available real-time PCR assays, including the qPCR of recommended by WOAH, are developed according to the sequences of European or American EIAV strains; however, the primers and probe sequences have low homology with Asian EIAV strains. To the best of our knowledge, no qPCR method capable of the well detection of Asian EIAV strains, especially Chinese EIAV strains, has been published to date. The development of a sensitive, specific, and rapid qPCR assay for the detection of the EIAV strains is therefore of great importance.

## INTRODUCTION

Equine infectious anemia (EIA) is caused by the EIA virus (EIAV), a lentivirus classified within the *Retroviridae* family, which infects equines. EIA has a worldwide distribution (1) and causes significant losses to the equine industry worldwide. EIA is one of the 11 equine diseases requiring compulsory notification of the World Organization for Animal Health (WOAH). The clinical manifestations of EIAV can be divided into more-or-less distinct phases. First, horses commonly experience an acute clinical episode, in which the main disease signs are fever, thrombocytopenia, and high-level viremia. Second, horses experience a chronic phase characterized by bouts of fever, thrombocytopenia, anemia, edema, depressed neurological reactions, and cachexia (1, 2). Finally, the equid enters a prolonged phase, the frequency of disease episodes diminishes, and the equid becomes an inapparent carrier. However, the inapparent carrier remains a potential reservoir of the virus for transmission (3). Therefore, early and accurate diagnosis of EIAV is critical in order to prevent the spread of EIAV.

The usual tests for this disease are pathological, serological, and molecular detection techniques. Pathological detection can diagnose EIA by immunohistochemical assays (4). However, immunohistochemical assays are complicated and time-consuming to perform. The serological tests include the agar gel immunodiffusion assay (AGID), which has been considered to be a gold standard in the detection of EIA by the WOAH, as well as enzyme-linked immunosorbent assays (ELISA) of gp45 and gp90 (5 – 7). However, serological detection techniques have certain limitations and are easily affected by the levels of antibodies. In the early stages of infection, the level of antibodies is low, leading to false negative results from serological tests. In addition, serological silence following EIAV infection has also been reported in a study, which showed that 18 positive horses in the PCR test were identified that were negative in a range of serological diagnostic tests and only one of these subsequently seroconverted during a 2-year observation period (8). Therefore, nucleic acid using PCR is considered to be a good complementary method to detect EIAV infections. The main techniques currently used for molecular detection are real-time PCR (9, 10). However, to date, real-time PCR-based detection methods have not always been effective where the sequences of EIAV strains have differed between countries due to geographic and other factors (11, 12). A few studies have reported that, in some instances, EIAV isolates were undetectable with the virus-specific primers recommended by the WOAH (WOAH Terrestrial Manual) (9, 13). Currently, the available real-time PCR assays are developed according to the sequences of European and American EIAV strains; however, their primers and probe sequences have low homology with Asian EIAV strains.

In this study, we developed a tat-gag-based real-time quantitative PCR (TG-qPCR) for the detection of EIAV by targeting the fragment between the tat and gag genes. We compared our TG-qPCR and the qPCR recommended by the WOAH by detecting Chinese and American EIAV in cell supernatants or synthesized plasmids containing the sequences of other EIAVs. The TG-qPCR performance was also evaluated in peripheral blood mononuclear cells (PBMCs) and serum samples.

## RESULTS

### Phylogenetic analysis

**Fig 1:**
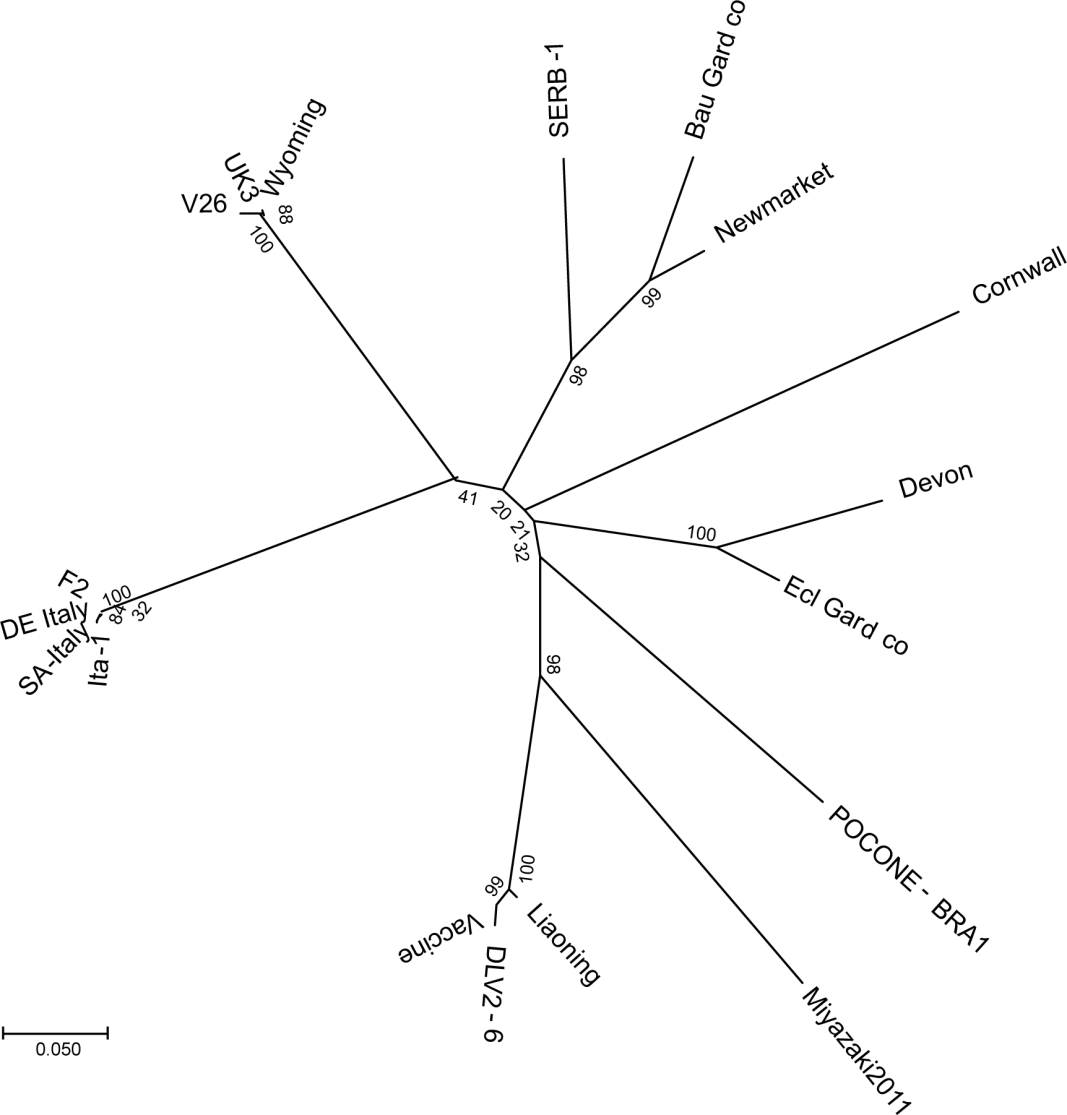
Sequence alignment of EIAVs from different regions. Phylogenetic analysis for the gag region of representative EIAVs from different regions using the maximum likelihood method based on the Tamura-Nei model with gamma distribution and invariant sites in the MEGA version 7.0 program. The 18 EIAVs include Bau Gard co (MK593462.1), Cornwall (MH580898.1), DE Italy (KM247554.1), Devon (MH580897.1), DLV2-6 (HM141920.1), Ecl Gard co (MK593463.1), F2 (JX480631.1), Ita-1 (EU240733.1), Liaoning (AF327877.1), Miyazaki2011-A (JX003263.1), Newmarket (MH580896.1), POCONE-BRA1 (MN560970.1), SA-Italy (KM247555.2), SERB-1 (MT338937.1), UK3 (AF016316.1), V26 (AB008197.1), Vaccine (AF327878), and Wyoming (NC_001450.1).

A phylogenetic analysis of gag sequences from 18 EIAVs was performed. The phylogenetic tree showed that although gag is the most conserved structural gene in the whole EIAV genome, it still has large genome variability (Fig. 1). The gag sequences of viruses from different geographical locations form eight separate clades. Interestingly, the 10 EIAVs from European are located in four separate clades. There are also some sequences from the same countries that fall into different clades, such as V26 and Miyazaki2011-A from Japan, Ecl Gard co and Bau Gard co from France, Cornwall, Devon, and Newmarket from England, which suggest that the gag gene has a relatively high variation.

**Fig 2:**
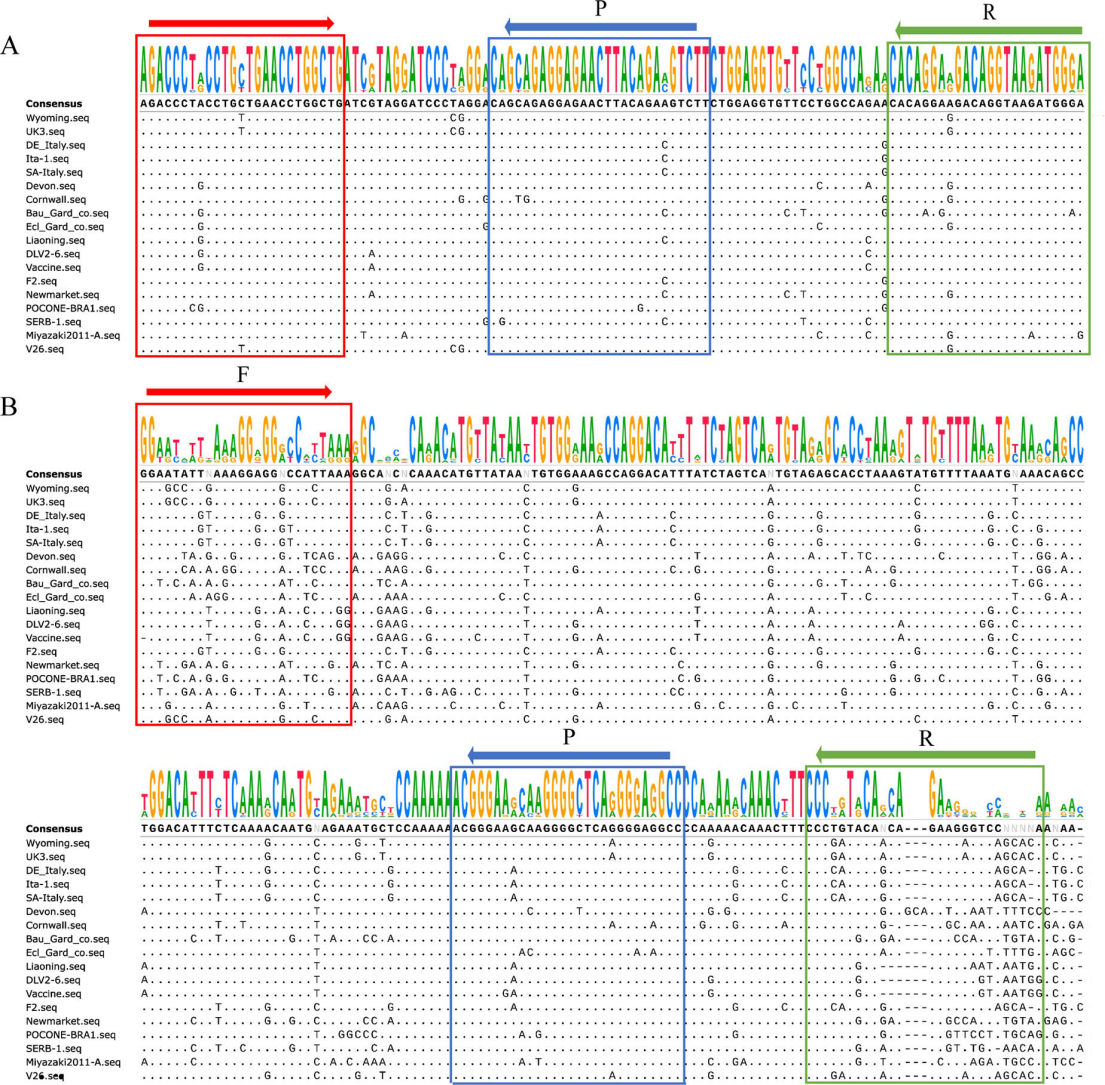
Sequence comparison of the DNA amplified fragments using TG-qPCR and qPCR of some EIAVs from different regions. The amplified DNA fragment sequences obtained using TG-qPCR (**A**) and qPCR (**B**) were aligned using EIAV sequences in different genomic regions. The F, R, and P letters stand for the positions corresponding to the forward primer, reverse primer, and probe sequences, respectively.

The sequences of the amplified DNA corresponding to the TG-qPCR and qPCR reactions were aligned (Fig. 2A and B). The alignment of EIAV nucleotide sequences demonstrated that the DNA sequences from the TG-qPCR were more conserved than those from the qPCR of recommended by WOAH.

### Analytical specificity of the TG-qPCR

The analytical specificity of the TG-qPCR was determined by testing the nucleic acids from EIAV, equine arteritis virus (EAV), EIV_H3N8_, EIV_H7N7_, EHV-1, EHV-4, *Salmonella abortusequi*, and *Streptococcus equi*. The TG-qPCR detected only the EIAV, but none of the others, suggesting that the TG-qPCR has high specificity.

### Analytical sensitivity of the TG-qPCR

**Fig 3:**
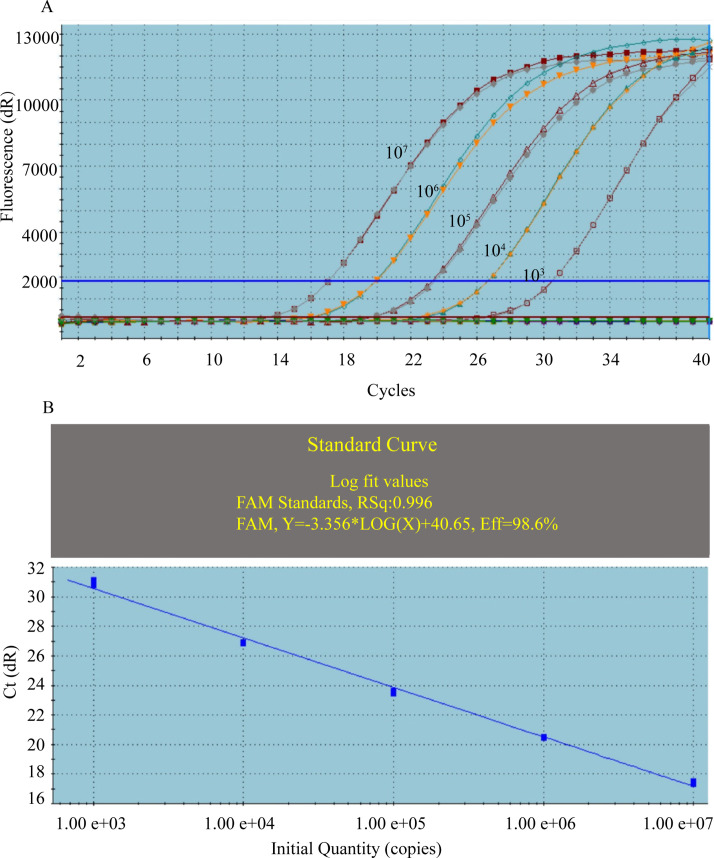
Amplification efficiency of TG-qPCR. (**A**) Amplification plots of TG-qPCR for the detection of standard plasmids. Copy numbers of standard plasmids ranged from 10^7^ to 10^3^ (left to right). (**B**) Corresponding standard curve. The curve shows DNA amplification plots with cycle threshold (Ct) values plotted against the logarithm of the input copy number. The standard curve has a slope of −3.356 and an RSq value of 0.996.

Serial 10-fold dilutions (10^3^–10^7^ copies) of the standard plasmids were tested using TG-qPCR in duplicate for each dilution. The coefficient of correlation (*R* ^2^) and amplification efficiency values were 0.996% and 98.6%, respectively (Fig. 3). RNA and proviral DNA copy numbers were calculated by the standard curve and then used for the analytical sensitivity assay. For more than five copies of proviral DNA and viral RNA from EIAV_UK3_ and EIAV_DLV2-6_ infected cells, the detection rates were 100% (Table 1). For testing 1 copy of proviral DNA extracted from either EIAV_UK3_ or EIAV_DLV2-6_ infected cells, the detection rates were 60% (12/20) and 65% (13/20), respectively. For testing 1 copy of viral RNA from EIAV_UK3_ and EIAV_DLV2-6_ infected cells, the detection rates were 60% (6/10). Therefore, the detection limit of the TG-qPCR was found to be 1 copy/reaction based on the Poisson distribution.

**TABLE 1:** Analytical sensitivity of TG-qPCR to detect proviral DNA and viral RNA from EIAV_UK3_ and EIAV_DLV2-6_ infected cells

Samples	Proviral DNA (copy/reaction)	No. tested	No. positive	Hit rate (%)	TG-qPCR (Ct)	Viral RNA (copy/reaction)	No. tested	No. positive	Hit rate (%)	TG-qPCR (Ct)
EIAV_UK3_	100	20	20	100	32.82 ± 0.34	100	10	10	100	33.04 ± 0.32
	50	20	20	100	33.64 ± 0.42	50	10	10	100	34.17 ± 0.30
	20	20	20	100	35.27 ± 0.41	20	10	10	100	35.69 ± 0.48
	10	20	20	100	36.12 ± 0.45	10	10	10	100	36.52 ± 0.49
	5	20	20	100	37.01 ± 0.42	5	10	10	100	37.55 ± 0.39
	1	20	12	60	38.48 ± 1.18	1	10	6	60	39.05 ± 0.62
	0	20	0	0	0	0	10	0	0	0
EIAV_DLV2-6_	100	20	20	100	33.82 ± 0.38	100	10	10	100	33.41 ± 0.32
	50	20	20	100	34.82 ± 0.53	50	10	10	100	34.34 ± 0.35
	20	20	20	100	35.35 ± 0.32	20	10	10	100	35.75 ± 0.42
	10	20	20	100	36.68 ± 0.38	10	10	10	100	36.52 ± 0.46
	5	20	20	100	37.65 ± 0.40	5	10	10	100	37.51 ± 0.50
	1	20	13	65	38.70 ± 0.80	1	10	6	60	39.12 ± 0.75
	0	20	0	0	0	0	10	0	0	0

### Reproducibility of the TG-qPCR

The intra-assay and inter-assay reproducibility of TG-qPCR were tested with 10-fold dilutions (from 10^0^ to 10^5^ dilutions) of the proviral DNA and viral RNA from EIAV_UK3_ infected cells (Table 2). The intra-assay reproducibility was tested in one run, and the coefficients of variation (CV) of Ct values were found to range from 0.32% to 2.5%. The inter-assay reproducibility was tested separately on 3 days, and the coefficient of variation of Ct values ranged from 0.1% to 2.2%.

**TABLE 2:** Intra-assay and inter-assay reproducibilities of TG-qPCR for the detection of proviral DNA and viral RNA from EIAV_UK3_ infected cells

	Proviral DNA					Viral RNA			
Dilution (fold)	Intra-assay		Inter-assay		Dilution(fold)	Intra-assay		Inter-assay	
	Ct	CV%	Ct	CV%		Ct	CV%	Ct	CV%
1	20.97 ± 0.22	1.0	20.89 ± 0.22	1.0	1	14.00 ± 0.21	1.5	14.14 ± 0.19	1.4
10^1^	24.09 ± 0.25	1.0	24.26 ± 0.27	1.0	10^1^	17.28 ± 0.23	1.3	17.43 ± 0.21	1.2
10^2^	27.40 ± 0.34	1.2	27.58 ± 0.38	1.4	10^2^	20.87 ± 0.18	0.85	20.77 ± 0.13	0.6
10^3^	31.64 ± 0.36	1.1	31.76 ± 0.38	1.2	10^3^	24.50 ± 0.08	0.32	24.42 ± 0.13	0.5
10^4^	35.15 ± 0.31	0.87	35.21 ± 0.29	0.84	10^4^	28.10 ± 0.09	0.33	28.26 ± 0.22	0.8
10^5^	38.86 ± 0.99	2.5	39.14 ± 0.85	2.2	10^5^	31.38 ± 0.25	0.79	31.50 ± 0.17	0.1

### TG-qPCR compared with the qPCR

Serial 10-fold dilutions of EIAV nucleic acids (proviral DNA and viral RNAs from EIAV_UK3_ and EIAV_DLV2-6_ infected cells) were prepared and tested in duplicate using the above methods. As shown in Table 3, EIAV nucleic acids could be detected using TG-qPCR assay. However, the qPCR was not able to detect proviral DNA and viral RNA of EIAV_DLV2-6_ infected cells but those of EIAV_UK3_ infected cells. Meanwhile, the maximum detectable dilution at which the proviral DNA and viral RNA of EIAV_UK3_ infected cells could be detected (10^3^) by qPCR was lower than that detected by TG-qPCR (10^5^).

**TABLE 3:** Detection limits of TG-qPCR and qPCR

Samples	Dilution (fold)	DNA		Dilution (fold)	RNA	
		TG-qPCR (Ct)	qPCR (Ct)		TG-qPCR (Ct)	qPCR (Ct)
EIAV_UK3_	1	20.94 ± 0.11	26.47 ± 0.35	1	14.00 ± 0.21	26.19 ± 0.01
	10^1^	24.35 ± 0.05	30.25 ± 0.02	10^1^	17.28 ± 0.23	29.77 ± 0.21
	10^2^	27.80 ± 0.53	33.36 ± 0.18	10^2^	20.87 ± 0.18	33.41 ± 0.06
	10^3^	32.12 ± 0.17	37.65 ± 0.19	10^3^	24.52 ± 0.08	36.56 ± 0.20
	10^4^	35.46 ± 0.13	No Ct	10^4^	28.11 ± 0.09	No Ct
	10^5^	39.86 ± 0.01	No Ct	10^5^	31.38 ± 0.25	No Ct
	10^6^	No Ct	No Ct	10^6^	35.05 ± 0.53	No Ct
EIAV_DLV2-6_	1	24.16 ± 0.56	No Ct	1	17.16 ± 0.11	No Ct
	10^1^	26.64 ± 0.23	No Ct	10^1^	20.43 ± 0.08	No Ct
	10^2^	30.06 ± 0.42	No Ct	10^2^	24.19 ± 0.00	No Ct
	10^3^	34.18 ± 0.04	No Ct	10^3^	27.98 ± 0.04	No Ct
	10^4^	37.48 ± 0.30	No Ct	10^4^	31.11 ± 0.04	No Ct
	10^5^	No Ct	No Ct	10^5^	34.22 ± 0.10	No Ct
	10^6^	No Ct	No Ct	10^6^	38.15 ± 0.10	No Ct

### The inclusivity of the TG-qPCR

The different templates representing as many sequences of EIAVs as possible were tested to evaluate the inclusivity of the TG-qPCR. The synthetic plasmids available were used as templates for TG-qPCR and qPCR amplification (Table 4). The qPCR was only able to detect the American EIAV_UK3_ strain and strains with phylogenetic relationship (Fig. 1), while all templates were detectable by our TG-qPCR. Therefore, the TG-qPCR is more suitable for global detection.

**TABLE 4:** Comparison of detection range between TG-qPCR and qPCR for detecting EIAVs from different geographic regions^*a*^

Templates^*a*^	TG-qPCR (Ct)	qPCR (Ct)
P-Wyoming	11.02	10.87
P-DE Italy	10.94	38.21
P-Ita-1	10.19	No Ct
P-SA-Italy	10.28	37.51
P-Devon	10.79	No Ct
P-Cornwall	10.21	No Ct
P-Bau Gard co	10.96	No Ct
P-Ecl Gard co	10.06	No Ct
P-F2	10.97	37.23
P-Newmarket	10.16	No Ct
P-POCONE-BRA1	11.23	No Ct
P-Miyazaki2011-A	11.52	No Ct
P-SERB-1	10.56	No Ct
P-V26	10.26	10.43
R-UK3	24.48	36.51
R-DLV2-6	25.71	No Ct
R-Liaoning	25.66	No Ct
R-Vaccine	29.98	No Ct

^*a*^ “P” indicates synthetic plasmid, and “R” indicates virus RNA from EIAV strains infected cells.

### Clinical performance of the TG-qPCR

The clinical performance of TG-qPCR was evaluated by testing seven PBMC samples collected from horses artificially immunized with the EIAV vaccine and six anti-EIAV sera from horses naturally infected with EIAV. For PBMC samples, the proviral DNA extracted from PBMCs was all judged positive by the TG-qPCR (Ct <39.74; Table 5). For sera samples, five of six samples were judged positive by the TG-qPCR (Ct <38.76), while one sample had no Ct value (Table 6). The second round of TG-qPCR amplification products of these samples were purified, connected to the PMD-18T vector, and sent to the Biotechnology company for sequencing. The sequencing results were consistent with the EIAV sequences published on NCBI. Therefore, the value of Ct <40 of TG-qPCR was judged positive by testing clinical samples.

**TABLE 5:** The performance of TG-qPCR for testing seven PBMC samples collected from horses artificially immunized with EIAV vaccine^*a*^

Animal ID	PBMC		Sera
	First round (Ct)	Second round (Ct)	Antibody
EIAV_2	33.10	9.42	+
EIAV_4	34.51	10.55	−
EIAV_5	39.74	10.44	+
EIAV_6	33.48	10.46	−
EIAV_9	35.77	10.39	−
EIAV_10	33.86	9.38	−
EIAV_11	35.92	10.12	−

^*a*^ “+” indicates a positive result, and “−” indicates a negative result in tests.

**TABLE 6:** The performance of TG-qPCR for testing clinical serum samples with antibodies against EIAV^*a*^

Field samples	TG-qPCR		ELISA
	First round (Ct)	Second round (Ct)	Antibody
1	No Ct	No Ct	+
2	38.76	10.86	+
3	38.58	10.42	+
4	37.89	9.38	+
5	37.20	8.31	+
6	37.96	9.67	+

^*a*^ “+” indicates a positive result in tests.

## DISCUSSION

EIA is an important infectious disease affecting the development of the global equine industry. In the absence of vaccines, it is very important to accurately and quickly diagnose EIA to prevent and control of the disease. Usually, diagnosis of EIA relies on the serological techniques AGID and ELISA. However, these serological diagnostic techniques have certain limitations and are affected by antibody levels. In the early stage of the disease, the antibody level in infected animals may be very low and may not be detectable by serological diagnostic techniques, resulting in false negative results. In most cases, seroconversion in equids is not easy to detect, and antibody levels remain very low following 45 or even 157 days of exposure to EIAV (14, 15). Moreover, serological silence has been reported in equids leading to antibodies being completely undetected, but this phenomenon is unlikely to be explained by host exposure to highly antigenically variant virus strains (8). Furthermore, it is possible that a serologically silent, occult form of EIAV infection exists similar to that described for hepatitis B virus (HBV), hepatitis C virus, and simian immunodeficiency virus where virus or viral nucleic acids are readily detectable but for reasons that have not been identified (8). If EIAV is not accurately diagnosed in its early stages, these equids may serve as potential sources of EIAV transmission (3, 15 – 17). Therefore, nucleic acid using PCR is considered to be a good complementary method to detect EIAV infections.

Assays based on PCR have revolutionized the diagnosis of infectious disease agents in many economically important plant and animal species, as well as in humans. Molecular diagnostic methods have been used previously for EIAV detection (17, 18). The primers of most PCR detection methods for EIAV were designed in the gag region (19 – 21). However, a comparison of the gag gene from European EIAV with that from North America and Asia shows that the gag gene is less well conserved than was previously believed (20). In our study, phylogenetic analysis showed that the gag gene sequences from 18 respective EIAVs form eight separate clades, with most of the North American and Asian sequences forming their own clades and the European forming four separate clades. In some cases, strains from the same country fall into different clades. The differences in the gag sequences mentioned above increase the difficulty of EIA nucleic acid detection. The currently available real-time PCR assays are not suitable for the identification of EIAV strains from some Asia-Pacific countries due to the mismatches of primers and probes against native viruses (13, 19). Therefore, researchers began to choose new target fragments to determine the sequences of these viruses using the sequences between 5’regions of the EIAV long terminal repeat and gag genes (9, 22). Unfortunately, to date, there is no universal real-time quantitative PCR method for the rapid clinical diagnosis of EIA. The main reason is that the EIAV strains are highly variable, and there is substantial genetic diversity among different geographically distinct isolates (23). It has been reported that the viral tat gene is likely to be conserved (24 – 26). In our study, the sequences of 18 representative viruses from different countries covering the tat and gag genes were aligned, revealing a relatively conserved fragment. A fragment between the tat and gag genes with even higher homology between the different EIAVs was noticed. A pair of primers and one probe were found according to the principles of primer and probe design (Fig. 2A), which were more conservative than the primer and probe sequences in the qPCR method recommended by WOAH (Fig. 2B).

Previous researcher established a TaqMan probe-based insulated isothermal RT-PCR with high sensitivity, an analytical sensitivity of 20 copies/reaction of viral RNA with a detection rate of 100% (27). In this study, we developed a TG-qPCR for the detection of EIAV, and our assay shows more sensitivity than the isothermal RT-PCR, with 100% detection rate at concentrations of 5 copies/reaction of proviral DNA and viral RNA. Furthermore, when testing for proviral DNA and viral RNA from EIAV_UK3_ infected cells, the maximum dilution detected by TG-qPCR was 100-fold higher than that detected by the qPCR assay recommended by WOAH (Table 3).

We assessed the performance of the TG-qPCR and the qPCR assay for the detection of Chinese EIAV (Liaoning strain, Vaccine strain, and others) and one American EIAV_UK3_ EIAV strains. The TG-qPCR was able to detect all the EIAV strains, while the qPCR was only able to detect the American but not Chinese EIAVs (Table 3). To further evaluate the inclusivity of the TG-qPCR, the synthetic plasmids were tested by the TG-qPCR and the qPCR, respectively. As shown in Table 4, the TG-qPCR was able to detect representative strains of EIAV from different countries; however, the qPCR was only able to detect the EIAV_UK3_, DE Italy, F2, SA-Italy, and V26.

It is currently almost impossible to obtain fresh PBMCs from horses suffering from EIA in China. The stored PBMCs collected 5 years ago from experimental horses were tested with our assay. Both the proviral DNA extracted from PBMCs and virus nucleic acid extracted from seropositive naturally infected horses can be detected by our assay. On one hand, the PBMC samples from the seven experimental animals immunized with attenuated vaccine all tested as EIAV proviral DNA positive, while only two of the corresponding sera tested positive for anti-EIAV antibodies (Table 5). This implies that TG-qPCR is better than ELISA in the early diagnosis of EIA. On the other hand, five of six viruses’ nucleic acid extracted from naturally infected seropositive horses were tested as positive using the TG-qPCR assay (Table 6). The discordant results of serological and molecular tests may be related to the fact that there is very little free virus in the serum, partially because these sera have been stored for a long period of time, and the degradation of the virus has occurred. On the whole, the above results suggest that this assay can be applied to detect EIAV proviral DNA from PBMCs or virus RNA from serum samples. Therefore, if considering reagents specifically for amplifying RNA is expensive, it is also an option of using reagents specifically designed for amplifying DNA to detect provirus in PBMC cells.

The TG-qPCR has better inclusivity for the detection of EIAV. The TG-qPCR assay could successfully detect viruses from China and the United States, as well as synthetic plasmids of representative strains from other countries, including England, Italy, France, Japan, Ireland, and the recently discovered isolates from Serbia and Brazil Pantanal (2020) (28, 29). The detection range of TG-qPCR has good coverage compared with the gag-based PCR method (9, 13, 18). Furthermore, we noticed that the published sequences of the EIAV strains recently detected in Brazilian Pantanal (2021) and Brazilian Northeast region (2022) (22, 30) are limited and not include the region of the forward prime of TG-qPCR; therefore, further study is needed for detecting these Brazilian epidemic EIAV strains.

In conclusion, we developed a TG-qPCR assay for the detection of EIAV with high specificity, sensitivity, reproducibility, and inclusivity. This assay could serve as a reliable tool for the rapid detection of EIAV in clinical PBMC and serum samples.

## MATERIALS AND METHODS

### Cells and viruses

Equine macrophage cells were isolated from the whole blood of healthy horses and cultivated at 37°C in an atmosphere of 5% CO_2_ in RPMI-1640 medium supplemented with 60% serum (fetal bovine serum and equine serum). Five days after the EIAV infection, macrophages were used to extract proviral DNA, and a cell culture supernatant was used to extract viral RNA.

EIAV_UK3_, EIAV_DLV2-6_, EAV, Equine influenza virus (EIV both H3N8 and H7N7 serotypes), equine herpes virus type 1 (EHV-1), equine herpes virus type 4 (EHV-4), *S*. *a*bortusequi, and *S. equi* were provided by the Harbin Veterinary Research Institute.

### Plasmid synthesis

The Chinese EIAV (Liaoning, Vaccine, and DLV2-6) and the America EIAV (UK3) were available in our laboratory, while the other viruses were not, so the specifically amplified sequences of those representative viruses were synthesized in order to evaluate the assay. The 18 whole-genome sequences of EIAV were downloaded from the National Center for Biotechnology Information (NCBI) and aligned, and the corresponding sequences of the amplicons corresponding to the TG-qPCR and qPCR were selected. These two fragments of each strain were linked with 10 bases and were synthesized by the Beijing Liuhe BGI Co. The P-Wyoming of synthetic plasmid was used as the standard plasmid for TG-qPCR. The concentrations of synthetic plasmids were measured on a NanoDrop 1,000 instrument, and the copy number of the standard plasmid was calculated using the standard formula (31).

### Phylogenetic analysis

The gag gene sequences were identified from the 18 EIAVs strains from Genbank and were aligned using Clustal W in Mega version 7.0 and were used to construct a phylogenetic tree. The phylogenetic analysis was performed in the Mega program, version 7.0 (32) using the Maximum Likelihood method and based on the Tamura–Nei model with a gamma distribution (five categories). Bootstrap values were determined over 1,000 replicates.

### Tat-gag-based real-time quantitative PCR

A total of 18 whole-genome sequences of EIAV were downloaded from NCBI and aligned using the Laser Gene sequence analysis package. After sequence alignment from the tat to the gag genes, a relatively conservative region was found (Fig. 2). Subsequently, specific primers (sense primer: 5′-AGACCCTRCCTGYTGAACCTGGCT-3′; anti-sense primer: 5′-YYCCATYTTACCTGTCYYCYTGTG-3′) and probe [TaqMan probe: 5’-(FAM) AAGACKTCTGTAAGTTCTCCTCTGCTG (MGB)−3’] were designed for the region between the tat and gag genes and were analyzed using the Oligo Analyzer 3.1 program (Integrated DNA Technologies) [https://www.idtdna.com/calc/analy zer (Fig. 2A)]. The TG-qPCR was performed using HiScript II U + One Step qPCR Probe Kit (Vazyme, China). Each TG-qPCR test comprised a 20 µL reaction consisting of 10 µL of 2 × One Step U + Mix, 1 µL of One Step U + Enzyme Mix, 6 µM of each primer, 4 µM of probe, after which 2 µL template was added, and finally, the reactions were made up to 20 µL with enzyme-free water. The reaction conditions were 55°C for 15 min, 95°C for 30 s, followed by 40 cycles of 95°C for 10 s and 60°C for 30 s.

The analytical sensitivity of the TG-qPCR was evaluated using 20 replicates of the EIAV proviral DNA and 10 replicates of RNA extracted from EIAV_UK3_ and EIAV_DLV2-6_ infected cells at copy numbers of 100, 50, 20, 10, 5, 1, and 0 per reaction. EIAV, EAV, EIV_H3N8_, EIV_H7N7_, EHV-1, EHV-4, *S*. *abortusequi*, and *S. equi* were tested to evaluate the specificity of TG-qPCR. Ten-fold dilutions of the proviral DNA and viral RNA from EIAV_UK3_ infected cells were tested, and reproducibility was evaluated by examining the CV of cycle threshold (Ct) values.

### Real-time quantitative PCR

A real-time qPCR assay recommended by the WOAH (18) was used as a reference method in this study. The specific primers and probe of this qPCR included: sense primer: 5′-GGAGCCTTGAAAGGAGGGCCACTAAA-3′, anti-sense primer: 5′-TTGTTGTGCTGACTCTTCTGTTGTATCGGG-3′, and probe [TaqMan probe: 5’-(FAM) ACGGGAAGCAAGGGGCTCAAGGGAGGCC (BHQ-1)–3’] (Fig. 2B). In addition, the amplification efficiency and sensitivity of qPCR were also assessed. The reaction systems and procedures were the same as for the TG-qPCR test.

### Clinical samples

To evaluate the reliability and clinical feasibility of TG-qPCR assay, both PBMCs and sera samples were used for testing for virus nucleic acid. PBMCs samples were collected from the whole blood of experimental horses immunized with the EIAV vaccine (33) and stored in our lab. Briefly, whole blood was centrifuged for 10 min at 1,000 × g, and the intermediate phase, corresponding to the fraction of mononuclear cells between the plasma and erythrocytes, was collected. The proviral DNA was extracted from these cells using the TIANamp Genomic DNA Kit (TIANGEN, China), according to the manufacturer’s instructions. Six anti-EIAV sera from horses naturally infected with EIAV were confirmed by our previously developed bELISA and stored in our lab (34). The nucleic acids were extracted using a Viral RNA Extraction Kit (YEASEN, China), according to the manufacturer’s instructions. The extracted nucleic acids were stored at −80°C until analysis.

### Sequencing

To validate the authenticity of the TG-qPCR results, sequence analysis of the EIAV-positive amplicons from clinical samples was performed. For samples with a TG-qPCR Ct value of more than 30, the amplified products were diluted 500 times as a template and amplified again, and the amplified products from the second round of TG-qPCR were fractionated in a 2% agarose gel and purified using a FastPure Gel DNA Extraction Mini Kit (Vazyme, China) prior to cloning into the pMD18-T vector, and the resulting plasmids were sent to the biotechnology company for sequencing.
